# Biological and Molecular Properties of Dengue 2 Strains Isolated during the DHF/DSS Cuban Epidemic, 1981

**Published:** 2007-06

**Authors:** Lester González, Mayling Álvarez, Delfina del Rosario, Alequis Pavón, Irina Prado, Joel Díaz, Luis Morier, María G. Guzmán

**Affiliations:** *Department of Virology, PAHO/WHO Collaborating Centre for the Study of Dengue and its Vector, Institute of Tropical Medicine “Pedro Kourí”, Havana City, Cuba*

**Keywords:** dengue, dengue fever, dengue hemorrhagic fever, DF, DHF, Cuba, biological properties, 1981

## Abstract

To study some biological and molecular properties of nine DENV-2 strains isolated during the 1981 Cuban epidemic, temperature sensitivity, viral plaque size, the kinetic of virus replication in newborn mice inoculated by intracerebral route, the influence of pH medium on virus-cell attachment phase and the restriction enzyme pattern were studied. Strains were classified in two patterns according to temperature sensitivity, plaque size, and virus replication in mouse brain and cell culture and restriction enzymatic pattern the changes observed differentiate clearly the strains isolated at the beginning and at the end of the epidemic suggesting that viruses with different characteristics circulated.

## INTRODUCTION

At the end of May of 1981, Cuba reported the first dengue hemorrhagic fever/dengue shock syndrome (DHF/DSS) epidemic in the American region. The epidemic, caused by the dengue 2 virus (DENV-2), was recognized simultaneously in three parts of the country and it was rapidly extended due to the high density of the vector, the mosquito *Aedes aegypti* (1). A total of 344 203 cases, 10 312 severe and very severe cases including 158 fatalities (101 children) in approximately four months were reported.

Contrary to expected, the case fatality rate increased by the end of the epidemic suggesting that when viral virulence apparently increased the agent was successively passed in the human host (2). This increase could be explained by the hypothesis that some of the population of antibodies against dengue 1 virus raised after natural primary infection (during the DENV-1 epidemic of 1977) react with neutralization determinants found on DENV-2 (3). Similar observations have been done later during the Cuban 1997 and 2001-2002 DENV-2 and dengue 3 (DENV-3) DHF/DSS epidemics (4, 5).

In this investigation some biological and molecular properties of the DENV 2 strains isolated in 1981 at two times-points of the epidemic (at the increase and at the decrease of the number of cases) are presented.

## MATERIALS AND METHODS

### Cell lines

*Aedes albopictus* cell line (C6/36HT ATCC CRL 1660) was grown at 33°C in Minimum Essential Medium with Earle’s balance salts (E-MEM) supplemented with 10% heated fetal bovine serum (HFBS), 1% non-essential amino acids and 1% L-glutamine solution 200mM. Maintenance medium was identical to the growth medium but supplemented with 2% of HFBS.

This cell line kindly provided by Javier Díaz (Departmental Laboratory of Medellín, Colombia) was employed to study the influence of medium pH in the virus attachment to the cell (6).

Baby hamster kidney cell line, BHK-21 clone 15 (sub-line of BHK-21 clones 13, ATCC CCL 10, (6), kindly provided by S. B. Halstead, University John Hopkins, New York, United States was employed to test the infectivity of viral strains, to study the viral plaque characteristics and the temperature sensitivity. Cells were grown at 37°C in E-MEM supplemented with 10% of HFBS, 10% L-glutamine solution 200 mM.

### Dengue 2 strains

Nine DENV-2 strains isolated in sera collected from patients with a clinical picture of dengue fever (DF) and DHF/DSS during the 1981 Cuban epidemic were included into the study. One strain was isolated from a DF case and five from DHF cases. The strains were isolated in newborn mice inoculated by intracerebral and subcutaneous routes (7) and kept at -80°C for more than 20 years. For the purposes of this study, strains were passaged once in newborn mice inoculated by intracerebral route.

Strains were divided in two groups:

Group A: Strains isolated from patients who developed the disease in June and July (period with a high number of dengue cases and the lower case fatality rate) (2);

Group B: Strains isolated from patients who developed the disease in August (period with a low number of dengue cases and the higher case fatality rate) (2).

Table 1 shows the characteristics of the studied strains including the viral plaque titre at the moment of the study. Table 2 shows the morbidity, the severity and the case fatality rate of the 1981 epidemic by months (2). Studied strains were grouped according the month of disease onset of the patients.

**Table 1:** DENV-2 strains studied in this investigation

Strain code	Clinic Classification	Month of onset of illness	Number of passage in mouse brain^a^	Plaque titre (log 10 pfu/ml)	Group

A15	DF	06/03/81	3	5,78	A
A35	DHF	06/11/81	3	5,82	A
A38	DHF	06/11/81	11	6,11	A
A115M2	DHF	07/17/81	2	6,66	A
A127	b	08/03/81	2	6,49	B
A129	DHF	08/08/81	2	6,23	B
A130	DHF	08/08/81	2	6,32	B
A131	b	08/11/81	2	6,59	B
A132	b	08/11/81	3	6,18	B

a All strains were isolated by intracerebral inoculation in newborn mice;

b Unknown.

**Table 2:** Case fatality rate, morbidity, severity of the 1981 epidemic and classification of studied strain according to month of disease onset (2)^a^

Months	Case fatality rate per 1000 patients	Morbidity	Severity	Groups of strains

June	0.39	96 684	19	A
July	0.42	183 443	34	A
August	0.83	48 315	44	B

a Case fatality = (fatal cases / total cases) × 1000; Severity = (severe cases / total cases) × 1000

### Virus titration

Viral plaque assay in BHK21, clone 15 was performed as previously described by Morens *et al*. (8) with some modifications (9). Briefly, 50 μL of virus dilution were added to 0.5 mL of BHK21 suspension (1.5 × 10^5^ cells) in each of three wells of a 24 well polystyrene plate. After 4 hours incubation at 37°C in 5% CO_2_, each well received 0.5 mL of 3% medium-viscosity carboxymethyl cellulose, made up Earle’s minimun essential medium with 10% HFBS. Plates were incubated for five days. After the incubation period, the medium was discarded by inverting plates. The plates were rinsed gently under tap water, fixed and stained using a solution of naphtol blue- black (1%) and acetic acid (6%). Plaques were counted and the virus titre was calculated.

### Temperature sensitivity and viral plaque characteristics

Studied strains were titrated as described above. For temperature sensitivity study, inoculated cells were incubated at 33°C, 37°C and 39°C. The average of the viral plaque titre was calculated at each tested temperature. Each experiment was repeated twice. All strains were tested simultaneously.

The size (in millimetres) and the morphology of the viral plaques were recorded as score.

### Virus replication in mouse brain

One- to two day-old suckling mice were inoculated intracerebrally with 0.02 ml volume containing 10^4^ plaque forming units (pfu) of the studied viruses. Daily, the brain of one animal was harvested and freezed at -80°C. Brains were homogenized to 10% suspension (weight/volume) in 199 media. The titre of the virus in the mouse brain was determined daily (8, 9). Deaths observed in the first 24-48 hours were not considered.

### Influence of the pH medium in the virus-cell attachment phase

C6/36 HT cells grown in plastic tubes were inoculated at a multiplicity of 0.01. The inoculation medium (E-MEM) was adjusted to pH6 and 7.5 with sodium bicarbonate. After one hour attachment at 33°C, the inoculum was eliminated; the cell monolayer was washed with maintenance medium without HFBS and 1 mL of maintenance medium was added. After 24 and 48 hours post-inoculation, supernatants of inoculated cells were collected and conserved at -80°C. Virus titration was performed as described above (8, 9).

### Enzymatic restriction analysis

Total RNA from 200 μL of homogenized of mouse brain infected with the study strains was extracted by Trizol (BDH, Germany) method, according to manufacture’s indications. DNA amplification was performed according to Lanciotti *et al*. (10) with some modifications (11). The region C-prM-M of the dengue genome was amplified. The restriction enzymes Hae III, Rsa I, Alu I and Hinf I and their corresponding buffers (Promega, UK) were employed in the enzymatic restriction assays. The protocol described by Rosario *et al*., 1997 was used (11). The digestion reaction consisted of 8 μL of the PCR product, 1 μL of the 10X digestion buffer (supplied with the enzyme (Promega, UK)) and 1μL of the enzyme (10 μ/mL). The mixtures were digested at 37°C for one hour and the products were electrophoresed on a 4% agarose gel prepared with buffer Tris Borate EDTA (TBE) 1X with 1.5 μL of etidium bromide (20 mg/mL). The digestion fragments were compared with a known molecular weight marker (100 bp DNA ladder, Promega, UK).

### Statistical analysis

The statistical package EPiInfo version of 1996 CDC, Atlanta, was used for the analysis of the results. The average of variables was compared by a parameter t-test. If *p*≤0.05 a statistically significant difference was considered.

## RESULTS

### Temperature sensitivity and viral plaque characteristics

**Figure 1:**
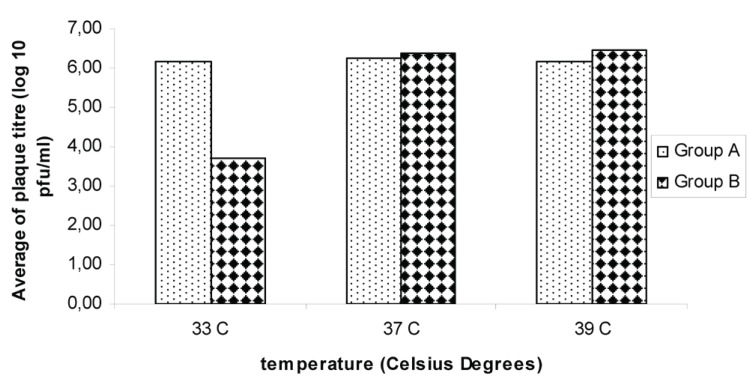
Effect of temperature on DENV-2 viral infectivity. ^a^Standard deviation; 33°C, *p*=0,001^*^; 37°C, *p*=0.15; 39°C, *p*=0.24.

Figure 1 shows the average of the viral plaque titre for the two groups of strains at the tested temperatures. A significant difference in viral titre (*p*=0.001) was observed among both groups at the temperature of 33°C. No differences were observed at 37 and 39°C temperatures.

**Figure 2:**
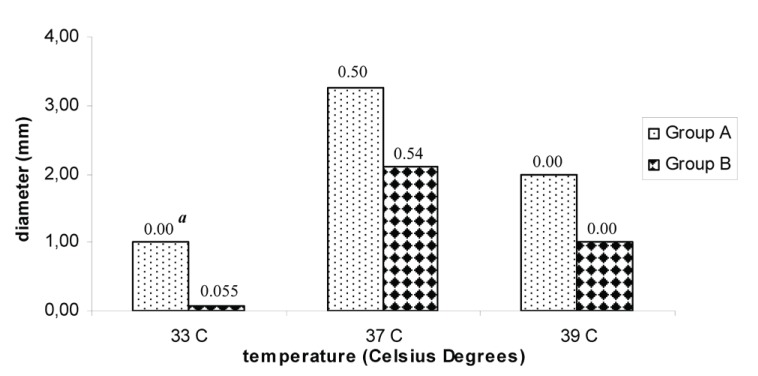
Average of plaque size of the DENV-2 studied strains at the three tested temperatures. a Standard deviation; 33°C, *p*=0.002^*^; 37°C, *p*=0.045^*^; 39°C, *p*=0.035^*^.

Compared to group B, group A strains showed significant higher viral plaque sizes at the three studied temperatures being highest at 37°C (*p*=0.045) and smallest at 33°C (*p*=0.002). Figure 2 shows the average of plaque size for both groups of strains.

**Figure 3:**
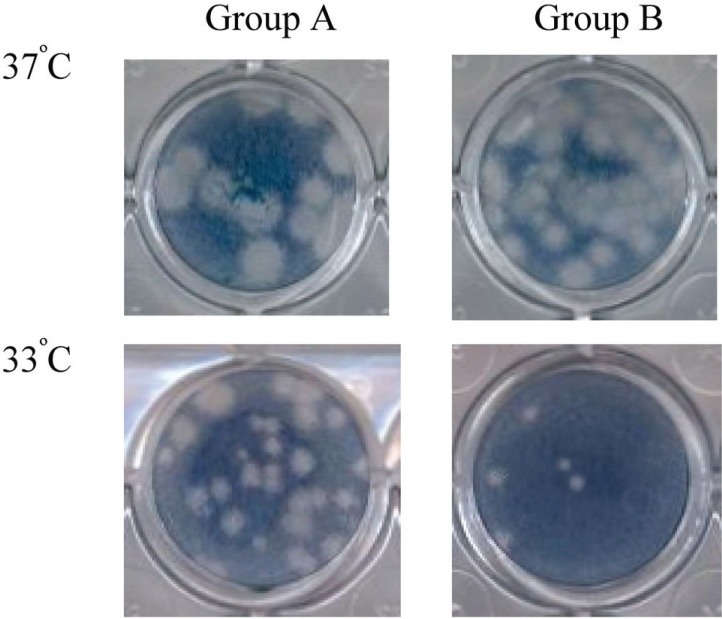
Viral plaques of DENV-2 studied strains at 37°C and 33°C.

Group B strains, presented a change in the plaque size and morphology; from large plaques at 37 and 39°C to small, confluent plaques or non plaques at 33°C. On the contrary, group A strains maintained the plaque morphology at the three temperatures tested in spite that plaques diminished in diameter at 33°C and 39°C (Figure 3).

### Virus replication in newborn mice brain

No illness was observed in the first three days after mice inoculation. On days four and five some signs of illness such as growth retard, paralysis of extremities and bristling were observed. At sixth day all mice died.

In time, an increased in the virus titre was observed for all studied strains. Viral titre was detected at days three (group A strains) and four (group B strains) after inoculation in all studied strains with the exception of the A15 strain in which titre of 6,0 × 10^1^ pfu/mL was detected 24 hours after inoculation. All strains showed the maximum viral titres at fifth day (Table 3).

**Table 3:** Plaque titre of the DENV-2 strains passaged in newborn mice brain

Strain Code	Log(pfu/mL)					
	Day 0	Day 1	Day 2	Day 3	Day 4	Day 5

A15	0	1.78	2.30	5.15	6.35	6.62
A35	0	0	0	2.96	4.96	6.52
A38	0	0	0	2.98	4.99	6.40
A115M2	0	0	0	2.60	4.28	6.28
A127	0	0	0	0	4.05	6.16
A129	0	0	0	0	4.10	6.28
A130	0	0	0	0	4.00	6.16
A131	0	0	0	0	4.18	6.51
A132	0	0	0	0	4.34	6.35

### Influence of pH of medium on virus-cell attachment

**Figure 4:**
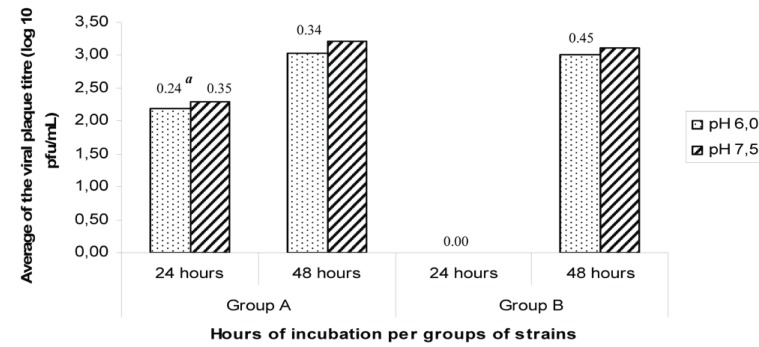
Influence of two medium pH on virus-cell adsorption. Average of DENV-2 plaque titre 24 and 48 hours after inoculation. ^a^Standard deviation; 24 hours pH6.0, *p*=0.00005**; 24 hours pH7.5, *p*=0.000001***; 48 hours pH 6.0, *p*=0.9765; 48 hours pH7.5, *p*=0.7698.

The average of viral titre of group A strains was significantly higher (*p*=0.00005** and *p*=0.000001***) than group B strains at the two medium pH tested. No virus replication (as detected by viral plaque titration) was observed for group B strains at 24 hours after inoculation. No significant differences were found 48 hours post-inoculation (Figure 4).

### Enzymatic restriction analysis

**Figure 5:**
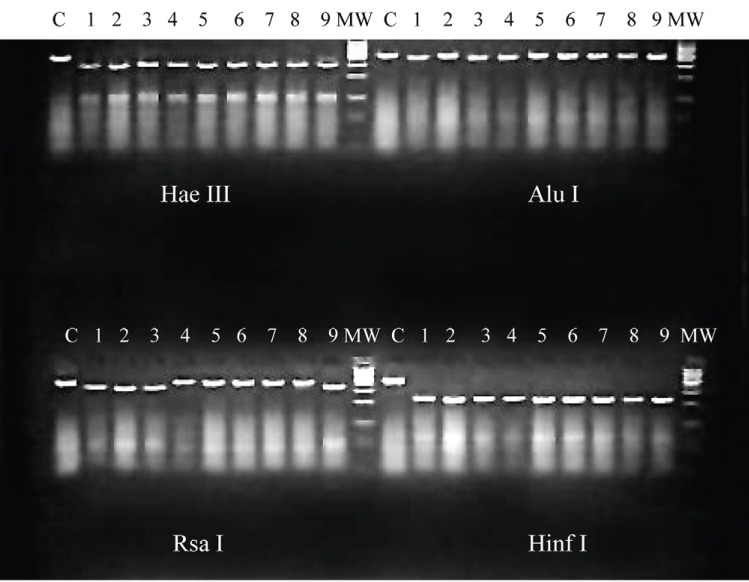
RFLP analysis on DENV-2 strains isolated during the 1981 Cuban epidemic. C, PCR product without enzyme treatment, 1, A15; 2, A35; 3, A38; 4, A127; 5, A129; 6, A130; 7, A131; 8, A132; 9, A115M2.

The restriction fragment length polymorphism (RFLP) patterns obtained when PCR products were digested with the enzymes Hae III, Alu I and Hinf I were identical. Two RFLP patterns consistent of two bands at different size were observed among the two groups of strains after treatment with Rsa I (Figure 5).

## DISCUSSION

During the 1981 and 1997 Cuban dengue epidemics, significant monthly increases were observed in the proportion of total cases that presented as DHF/DSS and in case fatality rates for both DF and DHF/DSS (3). Similar observations were done during the DENV-3 epidemic occurred in Havana in 2001/2002 (unpublished results). This observation could be explained by a rapid phenotypic change in a microorganism by the selection of neutralization-escape mutants allowing the selection of variant viruses in naturally immune populations.

Considering the importance of this epidemiological observation, the DENV-2 strains isolated during these epidemics are under research. Here we report some of the biological properties and the RFLP pattern of nine DENV-2 strains isolated in two time-points of the 1981 epidemic in an attempt to identify differences among them.

The 1981 DENV-2 strains were able to grow at 37°C and 39°C. A similar observation was previously done by Lopez *et al.* in the 2003 (5). These authors studied some DENV-2 strains isolated from DHF cases during the 1997 Cuban epidemic.

It’s remarkable that strains studied here form plaques and grow at a high titre at the highest temperature tested (39°C). Other authors (5, 12) have reported a reduction of plaque size and virus titre in DENV-2 and DENV-3 isolates grow at 39°C. As temperature sensitivity at 39°C is considered an attenuation marker, obtained results here suggest that the studied strains don’t show attenuation characteristics (13, 14).

Plaque size allowed identifying differences among both groups of viruses at the three studied temperatures. Particularly of interest was to note a diminishing in plaque size of group B strains at the three tested temperatures being smallest at 33°C.

An interesting observation of this study was the reduction in viral titre and size plaque at 33°C. This temperature has not been widely applied for dengue virus characterization. The results obtained in this study suggest that this temperature could be of interest to define or to identify differences among strains suggesting a higher or lower replication capacity.

Prolonged survival time of suckling mice after intracerebral inoculation of virus has also been reported as a possible marker of attenuation (14). All studied strains in this report killed inoculated mice at sixth day after inoculation however the kinetic of viral titre showed two patterns. Specifically, viral titre of group A was initially detected at day three post-inoculation and at day four in group B strains. These results suggest, that group B strains killed mice in a shorter period (24 hours) compared to group A strains (48 hours).

Considering that the observation period was five days, the presence of two well defined patterns at days three and four is of importance. It could be useful to test virus replication by using more sensitive systems such as Real time PCR allowing quantifying virus replication in the first hours after inoculation.

The influence of medium pH (6.0 and 7.0) at virus-cell attachment was tested for the first 24-48 hours after inoculation. Virus replication was detected in group A strains at both pH of medium, however, for group B strains, the virus replication was detected only at 48 hours post-inoculation. Rather than the influence of the medium pH at virus attachment, a delay in virus replication was noted similar to previous observations in inoculated newborn mice.

In general, the studied strains showed two patterns according to the biological properties studied here (Table 4). Characteristics of group A strains, isolated in the first weeks of the epidemic, suggest a better capacity of replication. These strains grew at the three studied temperatures, and replicated faster in inoculated mice and cell cultures independently of the medium pH. On the contrary, group B strains, isolated at the end of the epidemic, showed a higher restriction to grow at 33°C and a delay in the replication capacity in the first hours after inoculation however, these viruses were able to kill mice and to replicate to high titre in both mice and cell cultures suggesting a faster final progression.

**Table 4:** Summary of biological and molecular properties of Cuban DENV-2 strains isolated during the 1981 DHF/DSS epidemic

Groups	RFLP Pattern	Temperature sensitivity at 33°C	Plaque size reduction at 33°C	Delay on viral replication in newborn mice brain	Change of plaque morphology at 33°C and 39°C	Delay on viral replication at the first 24 hours according pH medium	
						pH 6.0	pH 7.5

A	A	No	No	No	No	+	+
B	B	Yes	Yes	Yes	Yes	-	-

The obtained results are not related to the system of virus isolation either the number of virus passage considering that the nine strains were isolated in newborn mice and that all but one presented 2-3 passages in this system at the moment of the study.

Also of interest was to note that RFLP pattern to *Rsa* I was different for both groups of strains suggesting the presence of some molecular change at the studied fragment that coding to C-prM and part of E protein. According to previous studies done by Rosario *et al*., in 1997 (15) strains A15 and A35 were included in a genetic variant closely related to New Guinea C strain, and others from Asian Southeast. Previously, Guzman *et al*. (16) and Sariol *et al.* (17) studied the E/NS1 junction segment and a fragment of the gene E of four DENV-2 strains. According to these studies, strains were classified as Asian genotype very close to old viruses such as the DENV-2 New Guinea strain.

In summary this study suggests that strains with different biological and molecular characteristics circulated during the 1981 epidemic. Specifically, the two observed patterns correspond with two different time-points of the epidemic, where case fatality rate changed from 0.39 to 0.83 and the severity (DHF/total cases × 1000) changed from 19 to 44 from June to August (Table 1) (2). The presence of viruses with different characteristics suggests the possibility of a virus change during the evolution of the epidemic. A similar phenomenon has been recently reported by Rodriguez-Roche *et al.* (4). These authors demonstrated nucleotidic and amino acidic changes at the non structural proteins of DENV-2 viruses isolated at different moments of the 1997 Cuban epidemic suggesting a clear pattern of viral evolution. The complete genomic study and testing of other biological properties of the 1981 viruses are in course in order to define if a similar phenomenon was observed during this epidemic.
